# Body mass index, body shape, and risk of nasopharyngeal carcinoma: A population‐based case–control study in Southern China

**DOI:** 10.1002/cam4.2027

**Published:** 2019-02-21

**Authors:** Ruimei Feng, Ellen T. Chang, Zhiwei Liu, Qing Liu, Yonglin Cai, Zhe Zhang, Guomin Chen, Qi‐Hong Huang, Shang‐Hang Xie, Su‐Mei Cao, Yu Zhang, Jingping Yun, Wei‐Hua Jia, Yuming Zheng, Jian Liao, Yufeng Chen, Longde Lin, Ingemar Ernberg, Guangwu Huang, Yi Zeng, Yi‐Xin Zeng, Hans‐Olov Adami, Weimin Ye

**Affiliations:** ^1^ Department of Cancer Prevention Center Sun Yat‐sen University Cancer Center Guangzhou China; ^2^ State Key Laboratory of Oncology in South China Collaborative Innovation Center for Cancer Medicine Guangdong Key Laboratory of Nasopharyngeal Carcinoma Diagnosis and Therapy Sun Yat‐sen University Cancer Center Guangzhou China; ^3^ Health Sciences Practice Exponent, Inc. Menlo Park California; ^4^ Stanford Cancer Institute Stanford California; ^5^ Department of Medical Epidemiology and Biostatistics Karolinska Institutet Stockholm Sweden; ^6^ Department of Clinical Laboratory Wuzhou Red Cross Hospital Wuzhou China; ^7^ Wuzhou Health System Key Laboratory for Nasopharyngeal Carcinoma Etiology and Molecular Mechanism Wuzhou China; ^8^ Department of Otolaryngology‐Head & Neck Surgery First Affiliated Hospital of Guangxi Medical University Nanning China; ^9^ Ministry of Education Key Laboratory of High‐Incidence‐Tumor Prevention & Treatment (Guangxi Medical University) Nanning China; ^10^ State Key Laboratory for Infectious Diseases Prevention and Control, Institute for Viral Disease Control and Prevention Chinese Center for Disease Control and Prevention Beijing China; ^11^ Sihui Cancer Institute Sihui China; ^12^ Cangwu Institute for Nasopharyngeal Carcinoma Control and Prevention Wuzhou China; ^13^ Department of Microbiology, Tumor and Cell Biology Karolinska Institutet Stockholm Sweden; ^14^ Beijing Hospital Beijing China; ^15^ Department of Epidemiology Harvard TH Chan School of Public Health Boston Massachusetts

**Keywords:** body mass index, body shape, case–control study, nasopharyngeal carcinoma, Southern China

## Abstract

Whether the association between body size or shape and nasopharyngeal carcinoma (NPC) risk exists or varies by age‐specific body size indicators is unclear. In a population‐based case–control study conducted in Southern China between 2010 and 2014, self‐reported height, weight, and body shape at age 20 and 10 years before interview were collected from 2448 histopathologically confirmed NPC cases and 2534 population‐based controls. Body mass index (BMI) was categorized according to the World Health Organization guidelines for Asian populations: underweight (<18.5 kg/m^2^), normal weight (18.5‐22.9 kg/m^2^), overweight (23.0‐27.4 kg/m^2^), and obese (≥27.5 kg/m^2^). Multivariate odds ratios (ORs) with 95% confidence intervals (CIs) were estimated using logistic regression. Furthermore, restricted cubic spline analysis was employed to examine nonlinear effects of BMI and body shape as continuous covariates. Underweight vs normal weight at age 20 years was associated with a 22% decreased NPC risk (OR, 0.78; 95% CI, 0.67, 0.90), whereas obesity was not significantly associated with NPC risk. Associations with BMI 10 years before the interview were similar. Having the leanest body shape at age 20 years, compared with the mode was not significantly associated with NPC risk (OR, 0.85; 95% CI, 0.62, 1.16), but having a larger body shape was associated with an elevated risk (OR, 1.25; 95% CI, 1.03, 1.52). Increasing BMI revealed positive trends with NPC risk. Despite some indication of significant findings, evidence for a strong association between BMI or body shape and NPC risk is still limited.

## 1 INTRODUCTION

Nasopharyngeal carcinoma (NPC) contributes relatively higher cancer burden in Southeast Asia, North Africa, the Arctic, and the Southern China than other areas in the world.^1^, ^2^ A growing number of studies suggest that the etiology of NPC is influenced by an interplay of genetics, Epstein–Barr virus (EBV) infection, and environmental factors.^3^, ^4^, ^5^ Body size and shape, which reflect the balance of energy consumption, physical activity, genetic factors, and environmental exposures, have a major influence on human health. Overweight or obesity, as measured by body mass index (BMI), is associated with increased risk of numerous malignancies, including colorectal cancer, oesophageal adenocarcinoma, renal cell carcinoma, postmenopausal breast cancer, as well as endometrial, thyroid, and liver cancer.^6^

In a limited number of studies, higher BMI has been linked with elevated risk of incident NPC, but reduced risk of prevalent NPC.^7^, ^8^, ^9^, ^10^, ^11^, ^12^ Because lower BMI predicts a worse NPC prognosis,^13^, ^14^, ^15^, ^16^ and weight loss may be a preclinical symptom of NPC, it is more appropriate to examine NPC risk in relation to body size in prospective studies with long follow‐up, or to assess body size in early adulthood, well before NPC onset. Another anthropometric indicator of interest is body shape, which can provide information on body fat distribution that is not captured by BMI. The Figure Rating Scale,^17^, ^18^ has been used to evaluate associations between body shape and various types of cancer,^19^, ^20^, ^21^ but as yet not NPC.

Insight into the impact of body size and shape on NPC risk can shed light on current understanding of disease etiology, and could potentially identify a new means to reduce the public health burden of this disease in endemic populations, where few primary prevention measures exist. Therefore, in our large‐scale, population‐based case–control study of NPC in Southern China, we evaluated the associations of BMI and body shape at age 20 and 10 years before interview with risk of incident NPC.

## 2 METHODS

### 2.1 Study design and setting

We conducted a collaborative, population‐based case–control study entitled “NPC Genes, Environment, and EBV” (NPCGEE) in the Zhaoqing area of Guangdong Province, and the Wuzhou and Guiping/Pingnan areas of the Guangxi Autonomous Region of Southern China between 2011 and 2014. All histopathologically diagnosed cases of NPC were ascertained by a rapid reporting system. Control subjects frequency matched to the expected age, sex, and geographic distribution of the cases were randomly selected from the dynamic population‐based registries. Eligible participants were those aged 20‐74 years, currently living in the study area during the recruitment period, and with no previous malignant disease or congenital or acquired immunodeficiency. Other details of the study design have been described previously.^22^, ^23^, ^24^, ^25^ Institutional review boards or ethics boards from all study centers approved this study. Eligible subjects granted written or oral informed consent.

### 2.2 Study eligibility

This analysis was restricted to participants aged more than 30 years (N = 5014) to allow for at least a 10‐year interval between assessment of body size and shape and diagnosis of NPC. Participants with missing information on height, weight, or body shape (N = 6), poor‐quality data (N = 1), or missing information on cigarette smoking, tea drinking, salt‐preserved fish consumption during 2000‐2002, and family history of NPC among first degree relatives (N = 25) were excluded, leaving 2448 cases and 2534 controls for the final analysis.

### 2.3 Assessment of body size, body shape, and other lifestyle factors

Data on individual lifestyle factors were collected by trained interviewers using an in‐person electronic questionnaire, as described previously. All participants reported their height in meters (m), weight in kilograms (kg), and self‐perceived body shape according to the Figure Rating Scale at age 20 and 10 years before the interview. The Figure Rating Scale uses a series of pictorial silhouettes to represent body shape on a scale from 1 (leanest) to 7 (largest) for males, and from 1 to 9 for females.^17^, ^18^

### 2.4 Statistical analysis

BMI was calculated by dividing weight (kg) by the square of height (m^2^), and then grouped into four categories according to the World Health Organization (WHO) guidelines for Asian populations: underweight (<18.5 kg/m^2^), normal weight (18.5‐22.9 kg/m^2^), overweight (23.0‐27.4 kg/m^2^), and obese (≥27.5 kg/m^2^).^26^ Body shape was classified into five categories (1, 2, 3, 4, or 5‐9). Category 3 was most commonly reported by controls both at age 20 and 10 years prior to the interview, and was therefore used as the reference category. Differences in the distribution of categorical BMI or body shape across potential confounders were compared using Chi‐square test or Fisher's exact method.

Unconditional logistic regression models were used to calculate adjusted odds ratios (ORs) and 95% confidence intervals (CIs) for associations between BMI or body shape at age 20 or 10 years before interview and NPC risk. Minimally adjusted ORs were controlled for the frequency matching factors: age at diagnosis/interview (30‐39, 40‐49, 50‐59, or 60‐75 years), sex (male or female), and residential area (Zhaoqing, Wuhzou, or Guiping/Pingnan). Additional potential confounders that were included in fully adjusted models were selected based on prior knowledge; these included education level (≤6, 7‐9, or ≥10 years), current housing type (building (concrete structure) or cottage (clay brick structure)/boat), current occupation (unemployed, farmer, blue‐collar, white‐collar, or other/unknown), cigarette smoking (ever or never), current tea drinking (yes or no), salt‐preserved fish consumption during 2000‐2002 (yearly or less, monthly, or weekly or more), and NPC among first degree relatives (yes, no, or unknown). In addition to evaluating associations with BMI or body shape at each of the two time points assessed, we also estimated ORs for change in BMI or body shape (grouped into three categories: shapes 1‐2, 3, or 4‐9) from age 20 to 10 years prior. Tests of the trend between the BMI, body shape and NPC risk were evaluated using the categorical BMI value or body shape score in the logistic regression models. Restricted cubic spline (RCS) logistic regression models with four knots (generally, the number of knots will be three to eight varied by the sample size, and the maximum number of knots supportable by our data can be four) were used to investigate any non‐linear effects of BMI or body shape on NPC risk by treating them as continuous covariates and treating the median level of BMI or body shape as the reference, *P* values for the overall associations between BMI or body shape and NPC risk, were calculated by the Wald Chi‐square test.

Analyses were performed with SAS version 9.4 (SAS Institute). The RCS analysis was conducted using the SAS Macro (*RCS_Reg* macro^27^). The two‐sided significance level for all statistical tests was 0.05.

## 3 RESULTS

### 3.1 Characteristics of control participants

Table 1 presents the distribution of categories of BMI at age 20 years across the baseline characteristics of 2534 population‐based controls aged more than 30 years. On average, participants older than 50 years had higher BMI than younger participants, and males had higher BMI than females. Participants with less education had higher BMI: the prevalence of overweight/obesity was 14.7% among those with ≤6 years of schooling, 11.1% among those with 7‐9 years, and 7.4% among those with ≥10 years. Ever smokers had lower BMI than never smokers.

**Table 1:** Characteristics of 2534 control participants aged more than 30 years old stratified by body mass index at age 20 years

Characteristics	Overall (N = 2534)	Body mass index at age 20 years (kg/m^2^)				*P* ^b^
		<18.5 (N = 509) N (%)	18.5‐22.9 (N = 1733) N (%)	23.0‐27.4 (N = 278) N (%)	≥27.5 (N = 14) N (%)	
Residential area						*0.055*
Zhaoqing	1282	243 (19.0)	912 (71.1)	122 (9.5)	5 (0.4)	
Wuzhou	648	146 (22.5)	419 (64.7)	78 (12.0)	5 (0.8)	
Guiping/pingnan	604	120 (19.9)	402 (66.6)	78 (12.9)	4 (0.7)	
Age at diagnosis/interview, years						***<0.001***
30‐39	375	77 (20.5)	256 (68.3)	41 (10.9)	1 (0.3)	
40‐49	892	168 (18.8)	635 (71.2)	83 (9.3)	6 (0.7)	
50‐59	733	145 (19.8)	503 (68.6)	82 (11.2)	3 (0.4)	
60‐75	534	119 (22.3)	339 (63.5)	72 (13.5)	4 (0.8)	
Sex						***0.018***
Male	1876	355 (18.9)	1315 (70.1)	197 (10.5)	9 (0.5)	
Female	658	154 (23.4)	418 (63.5)	81 (12.3)	5 (0.8)	
Educational level, years						***0.003***
≤6	937	181 (19.3)	619 (66.1)	130 (13.9)	7 (0.8)	
7‐9	1003	201 (20.0)	691 (68.9)	107 (10.7)	4 (0.4)	
≥10	594	127 (21.4)	423 (71.2)	41 (6.9)	3 (0.5)	
Current housing type^a^						*0.143*
Building (concrete) structure)	1963	380 (19.4)	1365 (69.5)	207 (10.6)	11 (0.6)	
Cottage/Boat (clay brick) structure)	571	129 (22.6)	368 (64.5)	71 (12.4)	3 (0.5)	
Current occupation						*0.146*
Unemployed	89	20 (22.5)	59 (66.3)	8 (9.0)	2 (2.3)	
Farmer	987	194 (19.7)	662 (67.1)	127 (12.9)	4 (0.4)	
Blue collar	864	164 (19.0)	617 (71.4)	78 (9.0)	5 (0.6)	
White collar	388	84 (21.7)	260 (67.0)	41 (10.6)	3 (0.8)	
Other/unknown	206	47 (22.8)	135 (65.5)	24 (11.7)	0 (0)	
Cigarette smoking						***0.008***
Never	1166	268 (23.0)	763 (65.4)	129 (11.1)	6 (0.5)	
Ever	1368	241 (17.6)	970 (70.9)	149 (10.9)	8 (0.6)	
Current tea drinking						*0.313*
No	1459	310 (21.3)	977 (67.0)	164 (11.2)	8 (0.6)	
Yes	1075	199 (18.5)	756 (70.3)	114 (10.6)	6 (0.6)	
Salt‐preserved fish consumption during 2000‐2002						*0.903*
≤Yearly	1857	370 (19.9)	1273 (68.6)	205 (11.0)	9 (0.5)	
Monthly	534	110 (20.6)	363 (68.0)	58 (10.9)	3 (0.6)	
≥Weekly	143	29 (20.3)	97 (67.8)	15 (10.5)	2 (1.4)	
Nasopharyngeal carcinoma among first degree relatives						***<0.001***
No	2422	485 (20.0)	1654 (68.3)	269 (11.1)	14 (0.6)	
Yes	70	15 (21.4)	48 (68.6)	7 (10.0)	0 (0)	
Unknown	42	9 (21.4)	31 (73.8)	2 (4.8)	0 (0)	

The *P* values were presented using the italic characters, and the bold characters indicated a statistical significance.

Current housing type includes building (concrete structure), cottage (clay brick structure) or boat.

*P* values for the difference across age at diagnosis/interview, and nasopharyngeal carcinoma among first degree relatives were derived using the Fisher's exact method, while the rest of *P* values were calculated using Chi‐square test.

Cases tended to be younger, to have a lower education level, more likely to live in a cottage (clay brick structure)/boat, to be blue‐collar workers, to be less likely tea drinkers, to be overweight at age 20 years, and to more likely have a family history of NPC (Table S1).

### 3.2 Risk of NPC in relation to BMI or body shape

Table 2 shows the ORs and 95% CIs for associations between BMI or body shape and risk of NPC. Because associations did not differ substantially between the minimally adjusted and fully adjusted models only fully adjusted ORs are presented in the text. Results did not vary appreciably between models with and without adjustment for individual indicators of socioeconomic status (ie, education level, current occupation, and current housing type) (data not shown).

**Table 2:** Odds ratios and 95% confidence intervals for nasopharyngeal carcinoma in relation to body mass index, or body shape

BMI and body shape	Cases (N = 2448) N (%)	Controls (N = 2534) N (%)	Minimally adjusted OR (95% CI)^a^	Fully adjusted OR (95% CI)^b^
BMI at age 20 years (kg/m^2^)				
<18.5	396 (16.2)	509 (20.1)	**0.77 (0.67‐0.89)**	**0.78 (0.67‐0.90)**
18.5‐22.9	1763 (72.0)	1733 (68.4)	1.00 (reference)	1.00 (reference)
23.0‐27.4	273 (11.2)	278 (11.0)	0.98 (0.82‐1.17)	0.95 (0.79‐1.15)
≥27.5	16 (0.7)	14 (0.6)	1.15 (0.56‐2.38)	1.19 (0.57‐2.49)
*P*‐trend				***0.016***
BMI at 10 years ago (kg/m^2^)				
<18.5	222 (9.1)	264 (10.4)	0.87 (0.71‐1.05)	**0.80 (0.66‐0.98)**
18.5‐22.9	1572 (64.2)	1593 (62.9)	1.00 (reference)	1.00 (reference)
23.0‐27.4	574 (23.5)	600 (23.7)	0.97 (0.85‐1.11)	0.99 (0.86‐1.13)
≥27.5	80 (3.3)	77 (3.0)	1.08 (0.94‐1.23)	1.01 (0.72‐1.41)
*P*‐trend				*0.225*
Body shape at 20 years				
Shape 1	83 (3.4)	106 (4.2)	0.86 (0.63‐1.15)	0.85 (0.62‐1.16)
Shape 2	915 (37.4)	963 (38.0)	1.00 (0.89‐1.14)	1.04 (0.92‐1.18)
Shape 3	1099 (44.9)	1158 (45.7)	1.00 (reference)	1.00 (reference)
Shape 4	293 (12.0)	254 (10.0)	**1.23 (1.02‐1.49)**	**1.25 (1.03‐1.52)**
Shape 5‐9	58 (2.4)	53 (2.1)	1.18 (0.80‐1.73)	1.09 (0.74‐1.62)
*P‐*trend				*0.125*
Body shape 10 years ago				
Shape 1	52 (2.1)	54 (2.1)	1.07 (0.72‐1.59)	1.06 (0.71‐1.58)
Shape 2	616 (25.2)	625 (24.7)	1.05 (0.92‐1.21)	1.03 (0.89‐1.18)
Shape 3	1140 (46.6)	1199 (47.3)	1.00 (reference)	1.00 (reference)
Shape 4	468 (19.1)	500 (19.7)	0.99 (0.85‐1.15)	0.98 (0.84‐1.15)
Shape 5‐9	172 (7.0)	156 (6.2)	1.18 (0.93‐1.49)	1.19 (0.94‐1.52)
*P‐*trend				*0.726*

The P values were presented using the italic characters, and the bold characters indicted a statistical significance.

Abbreviations: BMI, body mass index; CI, confidence interval; OR, odds ratio.

a Adjusted for age, sex, residential area.

b Adjusted for age, sex, residential area, education level, current housing type, current occupation, first‐degree family history of nasopharyngeal carcinoma, cigarette smoking, current tea drinking, and salt‐preserved fish consumption during 2000‐2002.

Control participants were more likely than NPC cases to be underweight at age 20 years (20.1% vs 16.2%) and 10 years prior (10.4% vs 9.1%), but the prevalence of overweight/obesity at both time points was similar between cases and controls. The presence of underweight vs normal weight at age 20 years was associated with a 22% decreased risk of NPC (OR, 0.78; 95% CI, 0.67, 0.90), whereas overweight (OR, 0.95; 95% CI, 0.79, 1.15) and obesity at age 20 years (OR, 1.19; 95% CI, 0.57, 2.49) were not significantly associated with risk, and there was an significant trend between continuous BMI level at age 20 years and NPC risk (*P*‐trend = 0.017). An inverse association was also detected with underweight 10 years before interview (OR, 0.80; 95% CI, 0.66, 0.98), but not with overweight or obesity 10 years prior (*P*‐trend = 0.23).

Control participants were slightly more likely than cases to report having been leaner (body shapes 1‐2) at age 20 years (42.2% vs 40.8%) and less likely to report having been larger (body shapes 4‐9) (12.1% vs 14.4%), whereas body shape 10 years prior was more evenly distributed between cases and controls. Compared with body shape 3, shape 1 at age 20 years was associated with a non‐significant decrease in NPC risk (adjusted OR, 0.85; 95% CI, 0.62, 1.16), whereas shape 4 was associated with a significant elevation in NPC risk (OR, 1.25; 95% CI, 1.03, 1.52), but we did not observe a statistically significant trend between continuous body shape score at age 20 or 10 years before interview and NPC risk.

**Figure 1:**
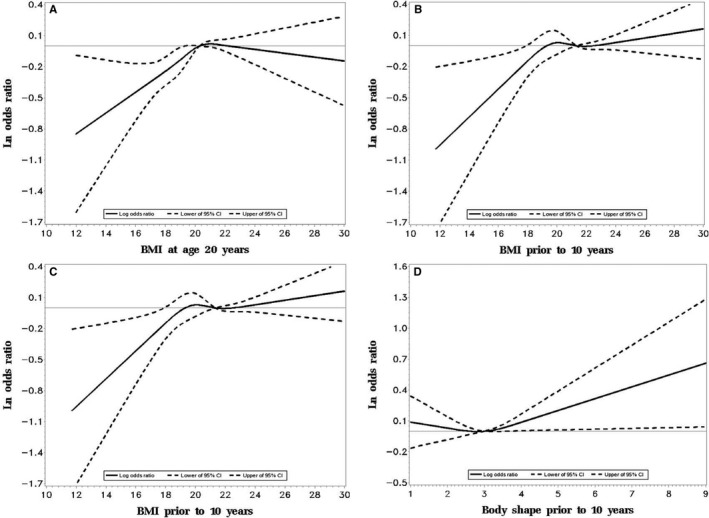
Ln odds Ratio and 95% confidence intervals for nasopharyngeal carcinoma in relation to body mass index and body shape (The restricted cubic spline analysis (RCS) for BMI (A and B) or body shape (C and D) (four knots) was adjusted for age, sex, residential area, education level, current housing type, current occupation, first‐degree family history of nasopharyngeal carcinoma (NPC), cigarette smoking, current tea drinking, salt‐preserved fish consumption during 2000‐2002). Abbreviations: BMI, body mass index; CI, confidence interval. (A and B) indicated the associations between continuous BMI at age 20 and 10 years before interview and the NPC risk by RCS, respectively; (C and D) indicated the associations between continuous body shape at age 20 and 10 years before interview and the NPC risk by RCS, respectively

These results were partly confirmed by the RCS shown in Figure 1. Specifically, with continuously increasing BMI at either age 20 years (Figure 1A) or 10 years before interview (Figure 1B), the ln OR increased from a negative value (indicating an inverse association with NPC risk) to a null value at the median BMI level (ie, ln OR = 0), then continued to increase with increasing BMI except for the BMI at age 20 years among the males (Figure S1). The tests for overall associations between continuous BMI at age 20 years (*P* = 0.001) or 10 years before interview (*P* = 0.034) and NPC risk were significant. Figure 1 also shows that with continuously increasing body shape category at age 20 years (Figure 1C) or 10 years before interview (Figure 1D), NPC risk was generally flat near unity (ln OR = 0) between body shapes 1 and 4, and sharply increased thereafter. However, only the associations with body shape at age 20 years (*P* = 0.041) was significant but not body shape 10 years before interview (*P* = 0.125). Similar trends were observed for both sexes (Figure S2).

### 3.3 Risk of NPC with change in BMI or body shape over time

Table 3 lists the ORs and 95% CIs for NPC risk in relation to BMI or body shape change between at age 20 years and 10 years before interview. Compared with participants with stable normal weight, participants who were underweight at age 20 years and underweight or normal weight 10 years before interview had a significantly decreased risk of NPC (OR, 0.74; 95% CI, 0.60, 0.92). Otherwise, no significant associations were detected with change in BMI over time, although a nonsignificant positive OR was observed for those who increased from normal weight at age 20 years to obesity 10 years prior (OR, 1.25; 95% CI, 0.79, 1.99). Compared with those who maintained a stable body shape 3, those who had a stable body shape 4‐9 were at significantly increased NPC risk (adjusted OR, 1.29; 95% CI, 1.04, 1.60). Otherwise, no other significant associations were detected for change in body shape over time.

**Table 3:** Odds ratios and 95% confidence intervals for nasopharyngeal carcinoma in relation to body‐mass index or body shape change

At age 20 years	At 10 years ago	Cases (N = 2448) N (%)	Controls (N = 2534) N (%)	Fully adjusted OR (95% CI)^a^
Body mass index change				
Underweight	Underweight	149 (6.1)	190 (7.5)	**0.72 (0.57‐0.91)**
	Normal weight	180 (7.4)	251 (9.9)	**0.74 (0.60‐0.92)**
	Overweight/Obesity	67 (2.7)	68 (2.7)	1.03 (0.72‐1.47)
Normal weight	Underweight	71 (2.9)	69 (2.7)	0.98 (0.69‐1.39)
	Normal weight	1293 (52.8)	1257 (49.6)	1.00 (reference)
	Overweight	353 (14.4)	371 (14.6)	0.95 (0.80‐1.13)
	Obesity	46 (1.9)	36 (1.4)	1.25 (0.79‐1.99)
Overweight/obesity	Underweight/Normal weight	101 (4.1)	90 (3.6)	1.09 (0.80‐1.48)
	Overweight	164 (6.7)	170 (6.7)	0.93 (0.74‐1.18)
	Obesity	24 (1.0)	32 (1.3)	0.70 (0.41‐1.22)
Body shape change				
Shape 1‐2	Shape 1‐2	542 (22.1)	548 (21.6)	1.03 (0.88‐1.21)
	Shape 3	353 (14.4)	399 (15.8)	0.98 (0.82‐1.18)
	Shape 4‐9	103 (4.2)	122 (4.8)	0.96 (0.72‐1.29)
Shape 3	Shape 1‐2	121 (4.9)	118 (4.7)	1.14 (0.85‐1.51)
	Shape 3	703 (28.7)	718 (28.3)	1.00 (reference)
	Shape 4‐9	275 (11.2)	322 (12.7)	0.89 (0.73‐1.08)
Shape 4‐9	Shape 1‐3	89 (3.6)	95 (3.8)	1.01 (0.74‐1.39)
	Shape 4‐9	262 (10.7)	212 (8.4)	**1.29 (1.04‐1.60)**

The bold characters indicted a statistical significance.

Abbreviations: BMI, body mass index; CI, confidence interval; OR, odds ratio.

a Adjusted for age, sex, residential area, education level, current housing type, current occupation, first‐degree family history of nasopharyngeal carcinoma, cigarette smoking, current tea drinking, and salt‐preserved fish consumption during 2000‐2002.

**Figure 2:**
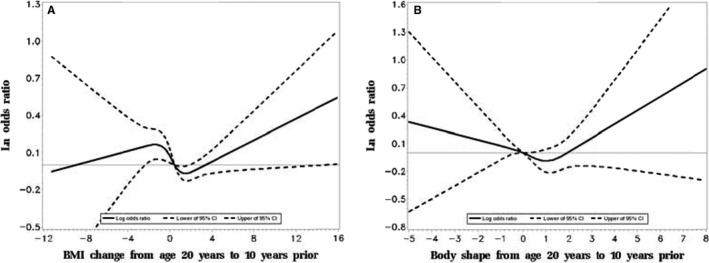
Ln odds ratios and 95% confidence intervals for nasopharyngeal carcinoma in relation to body mass index change or body shape change (The restricted cubic spline analysis (RCS) for BMI (A) or body shape (B) change from age 20 to 10 years before interview (four knots), was adjusted for age, sex, residential area, education level, current housing type, current occupation, first‐degree family history of nasopharyngeal carcinoma (NPC), cigarette smoking, current tea drinking, salt‐preserved fish consumption during 2000‐2002). Abbreviations: BMI, body mass index; CI, confidence interval. (A and B) indicated the associations between continuous BMI change and the bode shape change from age 20 to 10 years before interview and the NPC risk by RCS, respectively

Figure 2 shows the pattern of NPC risk in relation to continuous BMI or body shape change based on the RCS analysis. NPC risk increased above a 4‐kg/m^2^ gain in BMI between age 20 and 10 years prior (Figure 2A) (*P* = 0.043), whereas no clear trend was observed with change in body shape, due in part wide 95% CIs beyond a 2‐point change in either direction (Figure 2B) (*P* = 0.223).

## 4 DISCUSSION

To the best of our knowledge, our study is the first to assess the associations of both BMI and body shape with risk of NPC, as well as the first population‐based case‐control study of either association. We found that being underweight at age 20 or 10 years prior to interview was significantly associated with a decreased risk of NPC, with a suggestion of a positive trend between increasing BMI and risk of NPC. However, overweight or obesity per se was not significantly associated with NPC risk. For body shape, we found positive associations of NPC risk with a larger shape (4 vs 3) at age 20 years, and with maintenance of a larger body shape over time, but not with a larger body shape 10 years prior to interview.

Our results are generally consistent with those from a few previous studies that suggested a positive association between BMI and NPC risk. One prospective cohort study from Israel and two case–control studies from Zhejiang and Guangdong, China, showed that an increased NPC risk was associated with being overweight or obese vs normal weight or underweight,^8^ or that cases had a higher mean BMI than control subjects.^7^, ^9^, ^28^ However, a hospital‐based case–control study in Nairobi, Kenya, showed an inverse association with BMI, probably due to reverse causation.^12^ Although we did not observe a significant excess of NPC risk in association with being overweight or obese, the inverse association with being underweight and the positive association with larger body shape in our study are compatible with most of these earlier findings. However, when we recategorized BMI in our study according to the cutoffs used in earlier studies,^7^, ^8^ we found that higher BMI was not associated or only slightly, non‐significantly associated with increased risk of NPC. Most prior studies, with the exception of the cohort study, had limited sample size and insufficient control for confounding; None evaluated BMI in early adulthood, at least a decade prior to NPC diagnosis, and some results may have been influenced by disease symptoms or treatment.

The association of higher BMI with risk of numerous malignancies suggests shared carcinogenic pathways or an effect on a common underlying organ system, such as the immune system. Excess body fat is associated with elevated production of endocrine hormones, which may act on the carcinogenesis of NPC and other malignancies by regulating cell growth, proliferation, and apoptosis.^29^, ^30^, ^31^ Obesity may also suppress the immune response to infection,^32^, ^33^, ^34^ which in the context of NPC may influence the balance between viral latency and lytic replication.

Our study has several strengths, including its large size and rigorous protocol, and including quality control measures. In addition, we reduced the potential for reverse causation by assessing body size and shape in early adulthood, as well as 10 years before interview. However, the current study also has limitations. First, height, weight, and body shape were self‐reported, raising the possibility of misclassification and recall bias. In general, self‐reported height tends to be overestimated, but self‐reported BMI has been shown to be highly correlated with measured BMI.^35^ One study in Chinese adolescents reported a 0.809 Pearson correlation between measured and self‐reported values for BMI^36^; another study in the 5418 Chinese adolescents showed that Stunkard's current body size was a good indicator to assess weight status^37^; two studies reported high correlation between reported and measured weights in different time periods,^38^, ^39^ however, we were unable to identify any published validation studies for self‐reported BMI in Chinese adults. Second, we did not measure the ratio of waist circumference to hip circumference in our study. Instead, we assessed body shape using the Figure Rating Scale, which can reflect body fat distribution to some extent, as a body shape category >5 suggests abdominal obesity.^18^ Third, the low proportion of obesity limited our study's power to detect associations with BMI >27.5 kg/m^2^. The prevalence of obesity (0.7% among cases and 0.6% among controls) in our study is similar to that reported in a survey of the rural population in Guangdong Province (0.7%).^40^ The generally low BMI in the study area may be ascribed to the generally healthy diet in Guangdong and Guangxi, especially in rural areas.^40^ Fourth, we still lacked information regarding the EBV viral load level and histopathological subtypes, which limited the further stratified analyses by them. Last, we conducted a large number of significance tests, and some statistically significant findings might have arisen due to chance.

In conclusion, although our results provide some indication that lower BMI may be associated with reduced NPC risk, and that larger body shape may be associated with increased risk, the overall evidence supporting an association between BMI and NPC risk is still limited. For the best, the association with adiposity for the NPC risk can be regarded as modest. Further large‐scale studies, especially with prospective and repeated assessment of body size, are needed to explore the influence of these factors over the life course on NPC risk.

## CONFLICT OF INTEREST

All authors declare no conflicts of interest.

## Supporting information


**  **

**  **

**  **
