# Supplementary material for: Body mass index, body shape, and risk of nasopharyngeal carcinoma: A population‐based case–control study in Southern China
Source: Cancer Med. 2019 Feb 21;8(4):1835–44. doi: 10.1002/cam4.2027 (PMC6488148; doi:10.1002/cam4.2027)
Supplement: Supplementary file 3 [file CAM4-8-1835-s003.docx]

**Supplementary Table 1. Characteristics of 2,448 Nasopharyngeal Carcinoma Cases and 2,534 Controls Aged More Than 30 Years Old Stratified by Body Shape at Age 20 Years**

| **Characteristics** | **Cases**  **N=2,448**  **No (%)** | **Controls**  **N=2,534**  **No (%)** | ***P* ^a^** |
| --- | --- | --- | --- |
| **Residential area** |  |  | *0.264* |
| Zhaoqing | 1,234 (50.4) | 1,282 (50.6) |  |
| Wuzhou | 668 (27.3) | 648 (25.6) |  |
| Guiping/Pingnan | 546 (22.3) | 604 (23.8) |  |
| **Age at diagnosis/interview, year** |  |  | ***0.002*** |
| 30-39 | 425 (17.4) | 375 (14.8) |  |
| 40-49 | 912 (37.3) | 892 (35.2) |  |
| 50-59 | 683 (27.9) | 733 (28.9) |  |
| 60-75 | 428 (17.5) | 534 (21.1) |  |
| **Sex** |  |  | *0.939* |
| Males | 1,810 (73.9) | 1,876 (74.0) |  |
| Females | 638 (26.1) | 658 (26.0) |  |
| **Educational level, years** |  |  | ***0.004*** |
| ≤6 | 993 (40.6) | 937 (37.0) |  |
| 7-9 | 966 (39.5) | 1,003 (39.6) |  |
| ≥10 | 489 (20.0) | 594 (23.4) |  |
| **Current housing type ^a^** |  |  | ***<0.001*** |
| Building (concrete structure) | 1765 (72.1) | 1963 (77.5) |  |
| Cottage (clay brick structure)/Boat | 683 (27.9) | 571 (22.5) |  |
| **Current occupation** |  |  | ***<0.001*** |
| Unemployed | 75 (3.1) | 89 (3.5) |  |
| Farmer | 839 (34.3) | 987 (39.0) |  |
| Blue collar | 977 (39.9) | 864 (34.1) |  |
| White collar | 334 (13.6) | 388 (15.3) |  |
| Other/unknown | 223 (9.1) | 206 (8.1) |  |
| **Cigarette smoking** |  |  | *0.090* |
| Never | 1,068 (43.6) | 1,166 (46.0) |  |
| Ever | 1,380 (56.4) | 1,368 (54.0) |  |
| **Current tea drinking** |  |  | ***<0.001*** |
| No | 1555 (63.5) | 1459 (57.6) |  |
| Yes | 893 (36.5) | 1075 (42.4) |  |
| **Salt-preserved fish consumption during 2000-2002** |  |  | ***0.004*** |
| ≤Yearly | 1,795 (73.3) | 1,857 (73.3) |  |
| Monthly | 464 (19.0) | 534 (21.1) |  |
| ≥Weekly | 189 (7.7) | 143 (5.6) |  |
| **Nasopharyngeal carcinoma among first degree relatives** | |  | ***<0.001*** |
| No | 2,130 (87.0) | 2,422 (95.6) |  |
| Yes | 271 (11.1) | 70 (2.8) |  |
| Unknown | 47 (1.9) | 42 (1.7) |  |
